# Metabolic acidosis and 5-oxoprolinuria induced by flucloxacillin and acetaminophen: a case report

**DOI:** 10.1186/s13256-016-0964-x

**Published:** 2016-06-23

**Authors:** Charlotte Lanoy, Yves Bouckaert

**Affiliations:** Intensive Care Unit, Centre Hospitalier Universitaire (CHU) de Tivoli, Avenue Max Buset 34, 7130 La Louvière, Belgium

**Keywords:** Metabolic acidosis, Anion gap, Pyroglutamic acid, Sepsis, Antibiotic therapy

## Abstract

**Background:**

Frequent causes of high anion gap metabolic acidosis are well known: ethanol, methanol, and ethylene glycol intoxication; hyperglycemia; lactic or D-lactic acidosis; and impaired renal function. There are other causes, less frequent but also important. This report illustrates a rare case of a patient with increased anion gap metabolic acidosis due to a deficit of the γ-glutamyl cycle that led to 5-oxoproline (acid pyroglutamic) accumulation.

**Case presentation:**

An 82-year-old white woman was admitted to our intensive care unit because of septic shock caused by right knee methicillin-sensitive *Staphylococcus aureus*-induced arthritis. She was treated for 10 days with flucloxacillin and rifampicin and developed metabolic acidosis with high anion gap. Her test results for methanol, ethanol, ethylene glycol, and acetylsalicylic acid were negative. Her glycemia, lactate level, and renal function were normal. However, the result of a urinary assay for pyroglutamate was positive. We concluded that the patient had metabolic acidosis induced by accumulation of 5-oxoproline. We modified her antibiotic treatment, administered acetylcysteine, and her acidosis resolved.

**Conclusions:**

5-Oxoprolinuria (pyroglutamic acid accumulation) is a rare, probably underdiagnosed cause of transient metabolic acidosis with increased anion gap.

## Background

Metabolic acidosis is frequent in the intensive care unit (ICU) and can be divided into elevated or normal anion gap forms, depending on the levels of unmeasured anions in serum (Table 1). Frequent causes of elevated anion gap metabolic acidosis are well known: ethanol, methanol, and ethylene glycol intoxication; hyperglycemia; lactic or D-lactic acidosis; and impaired renal function. Other, less frequent causes are important to identify so that treatment approaches can be adapted appropriately. In this case report, we describe another cause of high anion gap metabolic acidosis due to 5-oxoproline (acid pyroglutamic) accumulation.

**Table 1:** Causes of high and normal anion gap metabolic acidosis

Increased anion gap	Normal anion gap
Lactic acidosis	Toluene ingestion (if late and if renal function is preserved)
Ketoacidosis: diabetes mellitus, starvation, alcohol use-associated	Diarrhea or other intestinal losses
Ingestion: methanol, ethylene glycol, aspirin, toluene (if early or if renal failure), diethylene glycol, propylene glycol	Type 2 renal tubular acidosis (proximal)
D-lactic acidosis	After treatment of ketoacidosis
Massive rhabdomyolysis	Carbonic anhydrase inhibitors
Pyroglutamic acidosis	Ureteral diversion
Chronic kidney disease	Chronic kidney disease and tubular dysfunction (but relatively preserved glomerular filtration rate)
	Type 1 renal tubular acidosis (distal)
	Type 4 renal tubular acidosis (hyperaldosteronism)

## Case presentation

An 82-year-old caucasian woman was admitted to the emergency room of our hospital with pyrexia (40 °C) and pain in her right knee and leg. She had a history of intraarticular injection of cortisone in the right knee 3 days before the admission. Her physical examination revealed erythema of the right lower limb with edema on palpation. The medical history of the patient was notable for severe aortic stenosis (estimated surface 1 cm^2^) with multiple episodes of hemodynamic pulmonary edema, for which the patient had refused any intervention; hypertension; type 2 diabetes mellitus; and a hiatal hernia. Her chronic treatment consisted of amlodipine, aspirin, olmesartan, furosemide, metformin, and omeprazole.

Blood testing conducted while the patient was in the emergency room showed a C-reactive protein (CRP) level of 47.5 g/L (normal <1 g/L), a bicarbonate level of 24 mmol/L (normal 22-30 mmol/L), and a hemoglobin level of 9 g/dl (normal 12–15 g/dl). Analysis of her knee fluid revealed the presence of methicillin-sensitive *Staphylococcus aureus*, for which she was treated with intravenous flucloxacillin (2 g six times per day), oral rifampicin (600 mg every day), and intravenous acetaminophen (1 g four times per day). Ten days after her admission, she developed encephalopathy associated with arterial hypotension at 70/50 mmHg that did not respond to crystalloid administration. The patient was transferred to the ICU, and blood gas analysis at her admission revealed increased anion gap metabolic acidosis with no elevated lactate level (pH 7.17, partial pressure of arterial carbon dioxide [PaCO_2_] 11.2 mmHg, partial pressure of arterial oxygen [PaO_2_] 122 mmHg, lactate 1.22 mmol/L).

Blood work conducted at the patient’s ICU admission confirmed that she had acidosis. She had a bicarbonate level of 4 mmol/L, a chloride concentration of 119 mmol/L, a sodium concentration of 145 mmol/L, and a potassium concentration of 3.6 mmol/L. The calculated anion gap was high at 22 (normal 8–12). The patient’s plasma osmolality was 283 mOsm/kg. Common causes of high anion gap metabolic acidosis, such as lactic acidosis, ketoacidosis, ingestion of toxic substances, and renal failure, were ruled out The patient’s blood glucose level was 174 mg/dl, and her renal function was normal (creatinine 0.87 mg/dl, normal 0.5–1.2 mg/dl; plasma urea 31 mg/dl, normal 17–42 mg/dl). A search for blood and urine ketones returned negative results, and the test results for levels of methanol, ethanol, and ethylene glycol by headspace gas chromatography and for levels acetylsalicylic acid with gas chromatography-mass spectrometry (GC-MS) were also negative. There was no indication of elevated D-lactate levels. In the context of treatment with flucloxacillin and acetaminophen, as well as the presence of severe sepsis, metabolic acidosis by accumulation of 5-oxoproline (pyroglutamic acid) was suspected. The diagnosis was confirmed by GC-MS analysis of urinary pyroglutamate (9789 mmol/mmol creatinine, normal level <14 mmol/mol creatinine).

The treatment consisted of stopping any medication that inhibits 5-oxoprolinase and causes glutathione deficiency. We replaced flucloxacillin with co-trimoxazole, which does not affect this enzyme. We also stopped the administration of acetaminophen and gave an infusion of acetylcysteine to replenish the patient’s glutathione stores.

The patient’s condition evolved favorably, and she was allowed to leave the ICU 8 days later. A blood gas sample taken at discharge showed regression of the metabolic acidosis (pH 7.53, PaCO_2_ 21.7 mmHg, PaO_2_ 80.2 mmHg, bicarbonate 18 mmol/L, calculated anion gap 8) and regression of the inflammatory syndrome (CRP 3.1 g/dl). The result of follow-up analysis of urinary pyroglutamate 15 days later was negative.

## Discussion

**Fig. 1:**
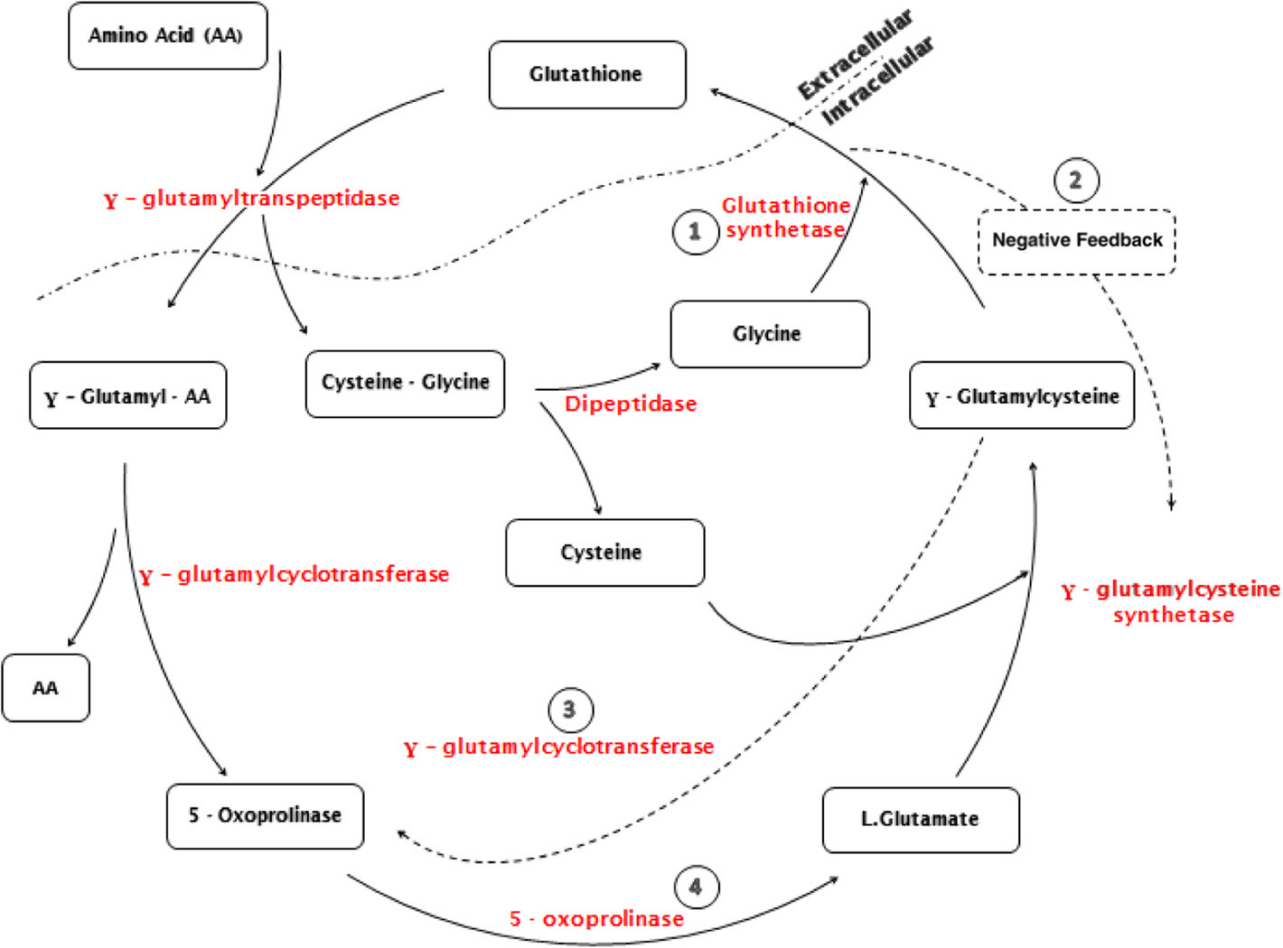
The γ-glutamyl cycle. Step 1: Glutathione synthetase deficiency induces a decrease in the levels of glutathione and an increase of γ - glutamyl cysteine. Step 2: Glutathione deficiency induces negative feedback in the γ-glutamyl-cysteine synthetase. Step 3: 5-oxoproline is produced by γ-glutamyl cyclotransferase from γ-glutamyl-cysteine. Step 4: 5-oxoproline is converted to L-glutamate by the 5-oxoprolinase, which is quickly saturated

5-Oxoprolinuria is a rare cause of increased anion gap metabolic acidosis. Pyroglutamic acid is dissociated in H^+^ and pyroglutamate. Increase in this unmeasured anion leads to high-anion-gap metabolic acidosis. This pathology is due to an anomaly of the γ-glutamyl cycle (Fig. 1) involved in the synthesis and metabolism of glutathione [1]. Glutathione is formed from glutamic acid, cysteine, and glycine; is a major cellular antioxidant; and is also involved in the regulation of apoptosis, cell cycling, and immunity. 5-Oxoprolinuria results most often from an enzymatic deficiency at the level of glutathione synthetase (Fig. 1, step 1) or 5-oxoprolinase (Fig. 1, step 4). The first catalyzes the transformation of γ-glutamylcysteine into glutathione, and the second catalyzes the conversion of 5-oxoproline (also called *pyroglutamic acid*) to L-glutamate [2].

Glutathione synthetase deficiency induces a decrease in the rate of glutathione production and an increase in γ-glutamylcysteine levels (Fig. 1, step 1). Glutathione deficiency produces negative feedback for the γ-glutamylcysteine synthetase (Fig. 1, step 2) and causes an increase of γ-glutamylcysteine [3]. As described by Schurmans *et al*. [4], this situation occurs more frequently in women (82 % of the cases reported) and during liver failure, malnutrition, sepsis, and treatment with certain drugs (including antiepileptic drugs and acetaminophen) that deplete glutathione [5]. Notable symptoms include encephalopathy, neurologic disorders, seizures, or hemolytic anemia [6].

On the one hand, accumulation of γ-glutamylcysteine is rare, because it is usually metabolized to 5-oxoproline, through a secondary pathway, by γ-glutamylcyclotransferase (Fig. 1, step 3). 5-Oxoproline is subsequently converted to L-glutamate by 5-oxoprolinase (Fig. 1, step 4). On the other hand, this enzyme is quickly satured, which can lead to the accumulation of pyroglutamic acid. This enzyme can be deficient due to a genetic abnormality (recessive autosomal) or inhibited by medications such as flucloxacillin (penicillin), ciprofloxacin (quinolone), or netilmicin (aminoglycoside) [7].

Our patient was a woman with sepsis who was treated symptomatically with acetaminophen. This situation probably led to glutathione deficiency and an increase of γ- glutamylcysteine. In addition, she received flucloxacillin for 10 days, which causes an increase in the rate of 5-oxoproline by inhibition of 5-oxoprolinase (see Fig. 1, step 4). These two mechanisms led to anion gap metabolic acidosis. The diagnosis was confirmed by the presence of increased urinary concentration of pyroglutamate [5]. In cases of associated kidney failure with decrease urine output (renal elimination of the pyroglutamate), a blood measurement can be performed instead of the standard urinalysis. Treatment consisted of discontinuation of the drugs that caused the problem. Administration of *N*-acetylcysteine to replenish stores of glutathione may be considered.

## Conclusions

5-Oxoprolinuria (pyroglutamic acid accumulation) is a rare, probably underdiagnosed cause of metabolic acidosis with increased anion gap. It is important to keep this possibility in mind for any patient with unexplained metabolic acidosis whose clinical features combine several risk factors (female sex, sepsis, malnutrition, liver failure, and certain medications), because the condition can be fatal and the treatment is simple. All the medications that can inhibit enzymes involved in the γ-glutamyl cycle and that consume glutathione must be discontinued. The administration of *N*-acetylcysteine may be recommended to replenish stores of glutathione.

## Abbreviations

CRP, C-reactive protein; GC-MS, gas chromatography-mass spectrometry; ICU, intensive care unit; PaCO_2_, partial pressure of arterial carbon dioxide; PaO_2_, partial pressure of arterial oxygen

## References

[1] Myall K, Sidney J, Marsh A. Mind the gap! An unusual metabolic acidosis. Lancet. 2011;377(9764):526. 10.1016/S0140-6736(10)61383-9 21296238

[2] Lawrence D, Bechtel LK, Charlton NP, Holstege CP. 5-Oxoproline–induced anion gap metabolic acidosis after an acute acetaminophen overdose. J Am Osteopath Assoc. 2010;110(9):545–51. 20876840

[3] Emmett M. Acetaminophen toxicity and 5-oxoproline (pyroglutamic acid): a tale of two cycles, one an ATP-depleting futile cycle and the other a useful cycle. Clin J Am Soc Nephrol. 2014;9(1):191–200. 10.2215/CJN.07730713 PMC3878708 24235282

[4] Schurmans W, Lemahieu W, Frans E. High anion gap metabolic acidosis: use the proper acronym, discard the red herrings and thou shall find the culprit. Clin Kidney J. 2014;7(3):320–2. 10.1093/ckj/sfu043 PMC4377761 25852900

[5] Verma R, Polsani KR, Wilt J, Loehrke ME. 5-Oxoprolinuria as a cause of high anion gap metabolic acidosis. Br J Clin Pharmacol. 2012;73(3):489–91. 10.1111/j.1365-2125.2011.04120.x PMC3370357 21988557

[6] Bernard L, Alastair C, Kelly C, Logan R. Transient 5-oxoprolinuria (pyroglutamic aciduria) with systemic acidosis in an adult receiving antibiotic therapy. Clin Chem. 1998;44(2):336–40. 9474033

[7] Fenves AZ. Kirkpatrick 3rd HM, Patel VV, Sweetman L, Emmett M. Increased anion gap metabolic acidosis as a result of 5-oxoproline (pyroglutamic acid): a role for acetaminophen. Clin J Am Soc Nephrol. 2006;1(3):441–7. 10.2215/CJN.01411005 17699243

